# Examining memory specificity and generalization contributions to false recall in DRM paradigm

**DOI:** 10.21203/rs.3.rs-9762596/v1

**Published:** 2026-05-28

**Authors:** Cheyna Warner, Lea Frank, Nash Unsworth, Dagmar Zeithamova

**Affiliations:** University of Oregon; University of Oregon; University of Oregon; University of Oregon

**Keywords:** DRM, false memory, individual differences, episodic memory, generalization

## Abstract

Why are some people more susceptible to false memory than others? Prior evidence indicates that false memories arise from a lack of specificity in memory representations. Additionally, false memory has been postulated to reflect a constructive generalization process that may be adaptive in nature. Here, we tested how individual differences in false memory relate to the ability to recall differentiating details (specificity) and the ability to integrate across related experiences (generalization) in a large sample of participants. False memory was assessed using the Deese, Roediger, and McDermott paradigm (DRM), where participants study lists of related words (e.g., sour, honey, tooth) and then often falsely recall non-presented critical lure (sweet). Participants completed the DRM paradigm along with multiple measures of memory specificity and generalization. Multiple regression revealed that higher memory specificity scores predicted greater true recall and lower false recall in DRM, supporting theories that emphasize the role of memory specificity in preventing false memories. In contrast, higher memory generalization predicted higher true recall but accounted for no unique variance in false recall when considering specificity. These findings inform current theories of false memory and indicate that false memories may stem from reduced representational specificity rather than adaptive abstraction.

## Introduction

Why are we susceptible to false memories? The framework for understanding the creation of false memories has been the center of much debate, with many theories pointing to a lack of specificity in memory trace as a key contributor to false memory. For example, the fuzzy trace theory postulates that we store memories through verbatim and gist traces (Brainerd & Kingma, 1984; Brainerd & Poole, 1997; Brainerd & Reyna, 2002). Verbatim traces are precise representations with specific details while gist is thought to reflect general meaning abstracted from experience. Both gist and verbatim traces are essential in aiding our ability to capture previously encountered stimuli (Reyna, 2012). While gist and verbatim traces are typically both stored, sometimes verbatim traces are inaccessible, causing people to rely instead on gist traces to inform behavior. Because the gist traces capture the meaning or theme of experiences, one may then falsely remember information that fits the gist, even when it did not happen (Brainerd & Kingma, 1984; Brainerd & Poole, 1997; Brainerd & Reyna, 2002; Webb et al., 2016).

Additionally, the spreading activation and source monitoring frameworks have also been applied to account for the emergence of false memories (Johnson, 1997; Johnson et al., 1993; Mitchell & Johnson, 2000). According to this view, a combination of two processes, spreading activation of related memory traces and a retrieval monitoring failure, give rise to false memory (Roediger et al., 2001). As related concepts activate each other in memory, encoding or retrieving information is typically accompanied by some level of activation spreading to related concepts, even if they themselves were not experienced during that particular event. Source misattribution or confusion can lead to false memory when an individual mistakenly attributes internally generated inferences, associations, or mental imagery to an externally experienced event. Studies linking false memory to poor source memory performance support this notion (Lindsay & Johnson, 1989).

Both of these mechanisms accept the assumption that memories are reconstructive and recognize the role of inference in memory error. Because key function of memory is to plan, imagine alternative scenarios, and predict the future (Schacter et al., 2007), it is often thought that false memories are features of a system optimized for flexibility and prediction rather than accuracy (Schlichting & Preston, 2015; Warren et al., 2014). As such, false memory may arise as a natural byproduct of an adaptive generalization process, being a flip side of our ability to go beyond direct experience to transfer our knowledge and infer new information (Pardilla-Delgado & Payne, 2017; Shohamy & Wagner, 2008; Varga et al., 2019). As generalization involves the formation of memories that integrate information across events, the distinctions between actually experienced events and derived knowledge may be left behind (Bowman et al., 2021).

The evidence for the notion that generalization and false memory go hand in hand comes from a couple lines of inquiry. For example, in category generalization paradigms, people typically confidently endorse as ‘old’ (previously encountered) never-seen category prototypes, which are central tendencies of categories abstracted from individual exemplars (Posner & Keele, 1970). In relational generalization paradigms, such as an associative inference task (AB & BC, therefore AC), people may misattribute information from one event (BC) to the related event (AB) after they successfully integrated the events (Carpenter & Schacter, 2017). Thus, on trial-by-trial level, successful generalization and inference may come at a cost to memory specificity. The relationship between generalization success and false memory may also exist at the level of individual differences. For example, individuals with ventromedial prefrontal pathology have both poor memory inference abilities (Spalding et al., 2018) and lower false memory tendency (Warren et al., 2014). Moreover, creative use of prior knowledge, as measured by divergent thinking tasks, is also associated with heightened rates of false memory (Thakral et al., 2021, 2023), indicating that constructive episodic processes may confer adaptive benefits at a cost of increased memory error. The hypothesis that a common memory integration process may lead to both generalization and false memory (Schlichting & Preston, 2015) was directly tested by Varga and colleagues (2019) using an individual differences approach across three tasks measuring false memory and new fact inferences. Although they found no relationship between false memories and generalization success across in Experiment 1, some aspects of the results could be interpreted as aligning with this hypothesis (Varga et al., 2019). Nevertheless, empirical evidence for generalization-false memory relationship is limited and mixed (Banino et al., 2016; de Araujo Sanchez & Zeithamova, 2023; Varga et al., 2019).

**Figure 1:**
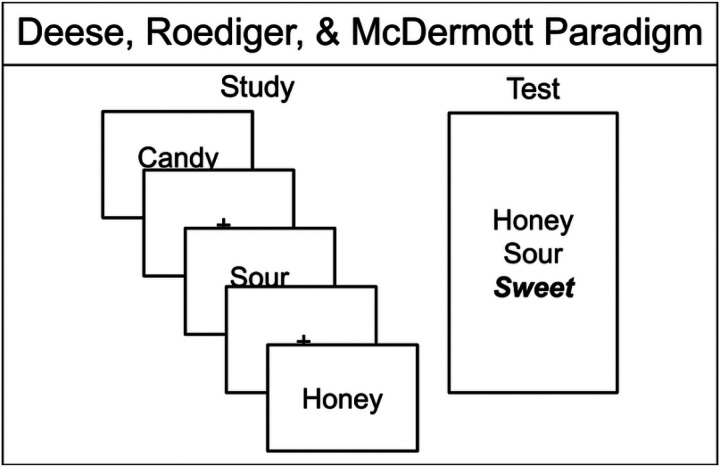
Deese, Roediger, and McDermott paradigm. Note. Participants are shown a series of words and later asked to recall or recognize the words they were presented. False memory occurs in the paradigm when participants erroneously recall or recognize semantically related, but never presented words. In this case, sweet (bolded) is an instance of false recall.

In the current study, we aimed to test the theory that generalization and false memory are the flip sides of the same coin (Pardilla-Delgado & Payne, 2017; Schlichting & Preston, 2015; Shohamy & Wagner, 2008; Varga et al., 2019) using an individual differences approach. Individual differences in false memory were examined using the Deese, Roediger, and McDermott (DRM) paradigm (Fig. 1), a foundational method of examining false memory in the laboratory. The task was designed to induce false memory, thought to result from an extraction of meaning (a gist or common theme) from a list of words (Deese, 1959; Roediger & McDermott, 1995). Participants study a list of related words, such as candy, tooth, sour, and honey. Importantly, one key word semantically related to studied words, such as ‘sweet’, is not presented during study but serves as a critical lure. Participants tend to spontaneously recall the critical lure during free recall or endorse the critical lure as “old” (previously studied) during a recognition test.

False memory in the DRM paradigm exhibits reliable individual differences (Ball, Robison, et al., 2022; Blair et al., 2002; Gallo, 2010; Salthouse, 2017; Salthouse & Siedlecki, 2007), which may stem from variability in memory strategies and their effectiveness during the encoding and retrieval phases of the task (Dewhurst et al., 2018; Unsworth, 2019). Multiple previous works have also examined memory performance across a broad range of tasks and have indicated that false memory in the DRM paradigm is driven, at least in part, by a lack of memory specificity (Ball, Robison, et al., 2022; Gallo, 2010; Salthouse, 2017; Salthouse & Siedlecki, 2007; Unsworth & Brewer, 2010; West et al., 2025, 2026).

While prior studies have convincingly linked individual differences in false memory in the DRM paradigm to a lack of memory specificity, the degree to which false memory in the DRM paradigm relates to memory generalization ability is less clear. In the current study, the DRM paradigm was administered in tandem with multiple memory tasks measuring memory specificity and generalization abilities to understand how false memory relates to the ability to remember differentiating details (specific memories) versus the ability to extrapolate across events (memory generalization). To measure specificity, we included tasks requiring memory precision, such as discrimination of old items from similar new items, as well as associative memory and source memory tasks. To measure generalization, we included both categorization tasks measuring similarity-based generalization and episodic inference tasks measuring relational generalization. For each memory construct of interest (memory specificity, memory generalization), we calculated a composite score across their four putative measures in order to more robustly estimate their theoretical relationship to false memory. Consistent with prior research, we hypothesized that individual differences in false memory rates would negatively correlate with individual differences in memory specificity, as predicted by theories postulating that DRM false alarms result from the lack of specificity in the memory trace. Secondly, we hypothesized that greater generalization ability would be associated with increased false memory, as predicted by theories postulating that false memory emerges as a “flip side” to adaptive generalization. Assessing individual differences in performance in these domains can help us gain a more robust understanding of how these memory processes operate and contribute to the constructive nature of memory.

## Methods

### Ethics and Consent to Participate

All procedures performed in studies involving human participants were in accordance with the ethical standards of the institutional research committee and with the 1964 Helsinki Declaration and its later amendments or comparable ethical standards. The study was approved by Research Compliance Services at the University of Oregon. All included participants provided their written informed consent to be included in the study.

### Participants

Two-hundred and thirty-one participants (157 female, 60 Male, 13 other; Mean age = 19.82; Range = 18–28) were recruited for the study from the University of Oregon campus and surrounding areas. Participants missing one or more memory measure were excluded from the present analysis, resulting in 202 participants included in all analyses. Participants provided written informed consent and received monetary compensation for their time.

### Study Procedure

Data were collected across 2–3 sessions conducted on different days, spaced most commonly 2 days apart. Each session lasted about 2 hours, with regular breaks. In addition to the DRM task, participants completed a battery of six tasks aimed at measuring memory specificity and generalization abilities. The tasks are described below in detail, but here we provide a brief overview. For generalization, we included two relational generalization tasks (associative inference, acquired equivalence) and two categorization tasks to measure similarity-based generalization (one cartoon animals and one with naturalistic faces). Two of the generalization tasks also provided measures of memory specificity: source memory test in the acquired equivalence task and face recognition test in the face categorization task. Two additional tasks focused on memory specificity only: object-color association task and a mnemonic similarity task. In total, we obtained for each participant four putative generalization measures and four putative specificity measures from the six tasks (Table 1). From these measures, we created one composite memory specificity score and one composite memory generalization score to relate to DRM true and false recall. The study was embedded into a larger individual differences study that collected additional cognitive and other measures not considered here. The order of the tasks was counterbalanced across participants.

### DRM Task

Participants completed the Deese-Roediger-McDermott paradigm (Fig. 1) to assess susceptibility to false memory (Deese, 1959; Roediger & McDermott, 1995). The task consisted of five blocks, each using one list of 10 semantically related words (e.g., candy, tooth, sour) associated with a non-presented critical lure (e.g., sweet). During the study phase, words were shown one at a time for 2 seconds each, with a 1 second intertrial interval. After all words were presented, participants completed a 2.5-minute math distractor task designed to prevent rehearsal. This task involved solving arithmetic problems with feedback provided on each response. Following the distractor, participants entered a free recall phase, where they had 45 seconds to type as many words from the studied list as they could remember. False memory was indexed by the frequency with which participants recalled the critical lure—a word strongly associated with the list but never actually presented. The main measures of interest in the DRM recall task were the proportion of presented words that participant recalled (true recall rate) and proportion of falsely recalled critical lures (false recall rate).

### Acquired Equivalence Task

**Figure 2:**
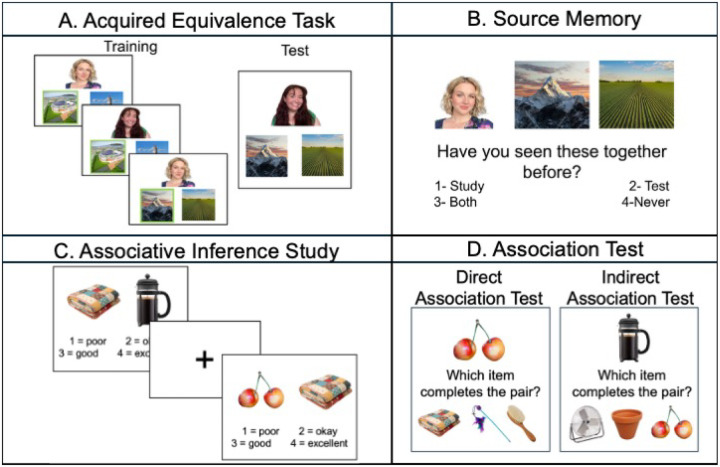
Associative inference and acquired equivalence tasks measuring generalization Note. **A. Acquired equivalence task**. Participants view a series of face-scene preference pairings and are asked to indicate the preferred scene for each face. Through feedback, participants learn face-scene preferences. At test, in addition to trained preferences, participants are tested on their tendency to generalize preferences between two faces based on a shared preference. **B. Source memory test following acquired equivalence task**. On each trial, participants indicated whether they had seen a particular face-scene-scene combination previously during study, test, both or never. **C. Associative inference study phase.** Participants are shown a series of image pairs and asked to generate stories tying each object together, rating the quality of the stories they generated. Some pairs overlap, affording an opportunity to link the related events**. D. Associative inference test.** After pair viewing, individuals are tested on their ability to remember direct (studied) associations and infer indirect associations.

An acquired equivalence task (Myers et al., 2003; Shohamy & Wagner, 2008) was used to measure spontaneous relational generalization, such as assuming that when two stimuli share one commonality, they may also share another. Here, we adapted a version that used overlapping face-scene pairing (Shohamy & Wagner, 2008). For example, after learning that Face 1 and Face 2 share a preference for Scene 1, participants learn that Face 1 additionally prefers Scene 2. The tendency to assign a preference for Scene 2 to Face 2 is a measure of generalization in this task (Fig. 2a). In the current study, we used a version that included a source memory test to provide a measure of memory specificity (de Araujo Sanchez & Zeithamova, 2023).

Study phase. Participants learned and were tested on face-scene pairs organized into 8 quadruplets (2 faces + 2 scenes). Before the study phase began, participants were exposed to each of the 16 faces in randomized orders (2s presentation, 1s fixation). After the exposure phase, participants completed study phase where they learned 24 face-scene pairs (3 pairs per quadruplet: Face 1-Scene 1, Face 2-Scene 1, Face 1-Scene 2). The order of the trials was scaffolded to support learning of the associations and generalization (de Araujo Sanchez & Zeithamova, 2023; Meeter et al., 2009), with each face-scene pairing repeated 8 times. On each trial, a face was shown at the top of the screen with two scenes below it (e.g., Face 1, Scene 1, Scene 3). Stimuli remained on the screen until the participant selected which scene the face prefers and then received feedback on their response (1 second). A given correct scene was always presented with the same foil scene to prevent learning during test and to enable source memory test (de Araujo Sanchez & Zeithamova, 2023).

Generalization test. After training, participants were tested on the 24 trained face-scene pairs and 8 new pairs (untrained Face 2-Scene 2 pairs). To obtain a more robust measure of memory and generalization, each directly learned association was tested twice and each generalization trial was repeated 4 times for a total of 80 trials. The correct response appeared equally frequently on the left and right side of the screen. Test was similar to the study portion, but no feedback was provided. Each trial was followed by a 1 second intertrial interval. Generalization was measured as the rate at which participants generalized Face 1 preferences to Face 2. For example, consistently choosing Scene 2 for Face 2, despite not being trained on that relationship, would reflect spontaneous acquired equivalence between the two faces (Face 1, Face 2) given their shared preference for Scene 1. Generalization scores for the subsequent individual differences analyses were derived from the subset of generalization trials for which directly learned associations were correctly remembered at every test repetition.

Source memory test. After the generalization test, participants completed a source memory test (Fig. 2b) to assess how well they can remember when they encountered each pairing (de Araujo Sanchez & Zeithamova, 2023). The source memory test consisted of the 36 face-scene-scene triplets encountered during both study and test (e.g., a studied trial consisting of cue Face 1, associated Scene 1, and lure Scene 3), 12 triplets encountered during test only (e.g., generalization trials such as cue Face 2, indirectly associated Scene 2, and lure Scene 4), and 36 recombined face-scene-scene triplets that were not presented together previously. On each trial, a face and two scenes were presented one on top of the other so the left-right organization of scenes was discarded and only scene identity was judged. Participants were asked when they had encountered the three images together: 1) only during the study portion, 2) only during the test portion, 3) during both study and test, or 4) never (the images were never shown all together before). The correct response for trained trials was “during both study and test”, the correct response for untrained trials was “during test only”, and the correct response for the recombined trials was “never”. The response “study only” was never correct but was included to avoid revealing the task structure from the response options (de Araujo Sanchez & Zeithamova, 2023). The test was self-paced with a 1 second intertrial interval. The specificity measure of interest from this test was overall source memory accuracy, or the ability to discern when information was encountered.

### Associative Inference Task

In the associative inference task (Preston et al., 2004; Zeithamova, Dominick, et al., 2012), participants encountered overlapping object pairs (A-B, e.g. coffee-blanket, blanket-cherries; Fig. 2c) and were then tested on the inference association between A-C items (e.g., cherries-coffee; Fig. 2d). The task consisted of an AB/BC observational training phase, an AC inference test, and an AB/BC associative memory test. On each training trial, participants were presented with a pair of object photographs and asked to generate a story or image to help them remember that the objects go together. Then, participants were asked to rate how vivid their story of the pairings is on a scale of 1–4 (1 being “poor” and 4 being “excellent”). Rating was included to encourage engagement and not considered further. Images were organized into 36 ABC triads. Each AB and BC pair was shown twice during training in a pseudo-random order, for a total of 144 training trials (72 AB, 72 BC).

Following study, participants were explained the structure of the stimuli they just encountered and were then tested on their ability to associate indirectly related objects (AC) through their shared association (A-B and B-C; Fig. 2d). On each trial, participants were shown an object A on the top of the screen and asked to pick which of three C options is the correct inference association based on the shared B object. This session consisted of all 36 A-C pairs repeated twice, for a total of 72 trials. Participants ability to correctly associate inference pairs is the main measure of interest from this task. The final test examined participants’ memory of the direct associations. For A-B pairings, object A was shown, and three B object options were given as choice. For B-C pairings, object B was shown, and three C objects were provided as choice. There were 144 trials to test participants on every pair. The final test was used to limit the analysis of AC performance to trials where both AB and BC from a given ABC triad were remembered.

### Faces Categorization Task

**Figure 3:**
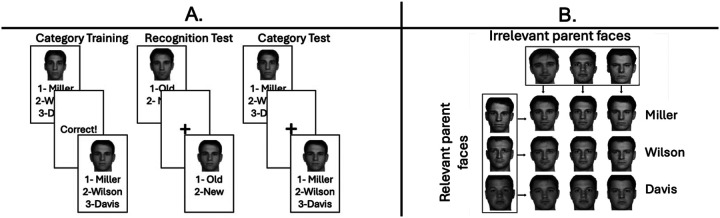
Face categorization and recognition task Note. **A. Face task.**Participants are shown images of faces and asked to sort them into categories. During training, participants receive feedback on their responses. Following training, participants complete a recognition task where they are asked to indicate if they have seen each face previously. Finally, participants were tested on learning the category structure by categorizing training and new faces without feedback. **B. Category structure for face categorization task.** Each face is a 50/50 blend of a category relevant face and a category irrelevant face, generating 9 face stimuli with shared categorizable features.

The face categorization task (Fig. 3a) was used as one measure of similarity-based generalization and included a recognition test as a measure of memory specificity (Ashby et al., 2020; Ashby & Zeithamova, 2022; Bowman et al., 2022). To generate similarity structure among the stimuli while maintaining realistic appearance, the face stimuli were generated by morphing pairs of “parent” faces that were real face photographs that originated from the Dallas Face Database. There was no overlap between faces used in this task and faces used in the acquired equivalence task. Each face was a 50/50 blend of one of three category relevant parents and one of three category irrelevant parent faces, for a total of 9 stimuli (Fig. 3b). Category relevant parent faces determined the category membership, with stimuli belonging into one of three families designated by last name (Miller, Wilson, and Davis). Category irrelevant parent faces affected physical similarity among faces but were not relevant for category membership.

The main task consisted of category training with the 9 training faces, an old/new recognition task, and a category test (Fig. 3a). In addition, participants passively viewed all training faces before the category learning task (4 repetitions of each of 9 training faces split across 2 blocks) and then again after category learning. The passive exposure phases were not critical for the present behavioral assessment but were included to make the task suitable for an accompanying neuroimaging study conducted with a subset of participants (not discussed here).

Category training. During training, participants were asked to learn the families (categories) that each training face belonged to through corrective feedback. On each trial, participants were asked to sort faces into one of three categories (Miller, Wilson, or Davis) and received feedback on their judgements. Training was split into two blocks separated by a self-paced break. Each block consisted of 16 repetitions of the 9 training faces, resulting in 144 trials per block and 288 training trials total.

Recognition. To measure participants’ memory for the specific faces they encountered during training, participants completed a self-paced old/new recognition task. Participants were shown a mixture of 9 training faces and 42 novel test faces, for a total of 51 recognition trials. New faces were generated by blending the original 3 category relevant parent faces with 14 new parent faces not used during training. For each face, participants made old/new judgments whether they had seen the face previously. Because the novel test faces were created from category-relevant parent faces blended with new faces, they bared resemblance to the old faces, requiring the participants to differentiate among similar faces. Corrected hit rate (hit rate minus false alarm rate) from the recognition test served as a measure of memory specificity. Although a combination of 9 old faces and 42 new faces creates an unbalanced design, the use of corrected hit rate ensures that participants cannot score well by simply responding “new” on every trial. Furthermore, our previous work (Ashby et al., 2020) as well as current results demonstrate that participants are able to reliability make correct judgments and perform well above chance.

Categorization test. Following recognition, participants completed a self-paced categorization task where they had to categorize old training and new test faces into the three categories learned previously. Same test faces were used in the categorization and recognition task. Categorization accuracy on the categorization test was the main generalization measure of interest from this task.

### Cartoon animal categorization

**Figure 4:**
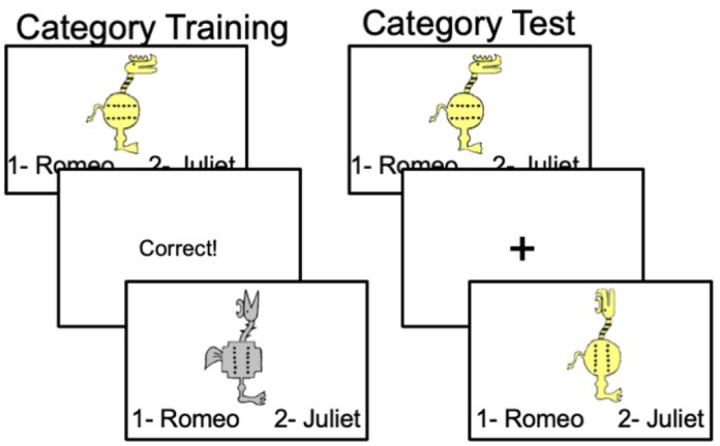
Animal categorization task Note. Participants are shown images of animals and are asked to sort them into categories. During training they receive feedback on their responses to promote category learning. Later, participants are tested on their knowledge of category structure by categorizing training (seen during training) and new animals without feedback.

A second categorization task measuring similarity-based generalization used cartoon animals, following procedures from (Bowman & Zeithamova, 2018). The animals varied along 8 binary dimensions (e.g., yellow v. grey, clawed v. webbed feet, forward or upward head orientation; Fig. 4). One cartoon animal was selected to serve as the category A prototype and the cartoon animal with the exact opposite features served as the prototype for category B. Cartoon animals that shared more features with the category A prototype than the category B prototype were members of Category A, and vice versa. Ambiguous stimuli equidistant from the two prototypes were not included in the study.

Training. Participants were trained on 8 animals that were each 2 features away from their category prototype. Our prior work showed that this training structure encourages the creation of generalized category representations rather than exemplar memorization (Bowman & Zeithamova, 2018). Training was split across 4 blocks, with 7 randomly ordered repetitions of each animal per block (224 total trials). For every trial, participants were shown a cartoon animal on the screen for 1 second and were then asked to sort it into one of two categories. After making judgements, they received feedback on their response followed by a fixation cross. Following training, participants completed a category generalization task to test how well they learned the category structures. This test included the 8 training animals, both category prototypes, and 48 new animals (8 new animals per each distance from each prototype). The training stimuli and prototypes were both tested twice while all other stimuli once. Test was self-paced and no feedback was given. Categorization accuracy at test served as a key generalization measure.

### Modified mnemonic similarity task

**Figure 5:**
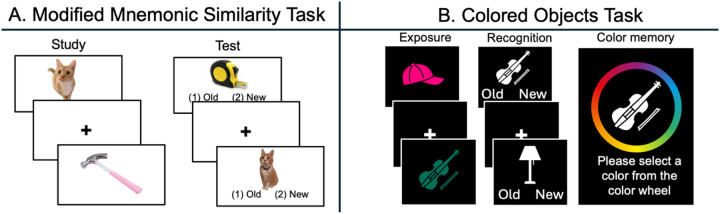
Memory specificity tasks Note. **A.** Modified **mnemonic similarity task**. Participants are shown a series of images. Following study, they are asked to identify if images were old or new. **B. Colored objects.**Participants view a series of colorful objects and are then asked to make old/new judgements. Following recognition, participants engage in a color memory task where they must indicate what color each object was previously presented in on a color wheel.

In addition to two specificity measures obtained from the tasks described above (source memory from acquired equivalence task, face recognition from face categorization task), we included two tasks directly focused on measuring memory specificity. Participants completed an adapted version of the mnemonic similarity task (Bakker et al., 2008; Lacy et al., 2011; Stark et al., 2019; Fig. 5A). During the study phase, participants were asked to remember 48 images (24 unique animals, 24 unique tools, to avoid overlap with stimuli used in other tasks). The images were presented on a screen one at a time for 2 seconds, separated by a 1 second fixation cross. Participants then completed a recognition test, where they were asked to identify 48 images as old or new. Test images consisted of 24 studied images and 24 lure images (e.g., a different hammer, a different cat, etc.). The test was self-paced, and each trial was separated by a fixation cross. Corrected hit rate (hit rate for studied items - false alarm rate to lures), was used as the measure of memory specificity in this task.

### Colored Objects Task

The colored object task measures how precisely one remembers a color of an object (color-object associative memory), in addition to measuring object recognition memory (Brady et al., 2013; Cooper & Ritchey, 2019). During the study phase, participants passively viewed 50 images of objects that were each one solid color (Fig. 5b). Following study, participants completed an object and color recognition task. On each test trial, participants saw one of 75 objects (50 old, 25 new) presented without color. They were first asked to make an old/new judgement for the object, followed by a color memory test (Fig. 5b). Participants indicated what color each presented object was by clicking on the corresponding color on the wheel. Each location on the color wheel was internally represented as a degree of angle (a number between 0 and 360 degrees) and we measured the absolute error (in degrees of angle) between the true color and the reported color on the color wheel. The smallest distance between the true color and the indicated color on the color wheel was used such that the error was always between 0 and 180 degrees. For example, if the true color was 0 degrees on the color wheel, both the response corresponding to 30 degrees and the response corresponding to 330 degrees on the color wheel would be scored as an error of 30 degrees, as both are 30 degrees right or left from zero on the color wheel. Color memory precision was the main measure of memory specificity obtained from this task. Because smaller errors indicate better memory in this task, we inverted the sign after z-scoring this measure for subsequent individual differences analysis. This ensured that participants with the smallest errors were assigned highest positive scores, aligning the object color memory precision measure with other memory performance scores where higher scores indicate better memory. We also calculated old/new object recognition score but did not to include it in the overall memory specificity composite score as it was highly correlated with the color memory, which would lead to a greater weight of this task in the overall composite score. However, we verified that inclusion or exclusion of the object recognition score does not alter any conclusions of this manuscript.

### Overview of statistical analysis

For the main analysis, two composite scores were created, one indexing individual differences in generalization ability and one indexing individual differences in the ability to remember specific details. The composite scores were calculated by z-scoring each a priori measure of interest and averaging those z-scores. Prior to calculation of composite scores, performance was determined to be above chance, and each measure was evaluated for reliability. The generalization composite score included four measures: cartoon animals categorization accuracy, face categorization accuracy, acquired equivalence score (tendency to generalize preferences across related faces in the acquired equivalence task), and associative inference score. The specificity composite score also included four measures: corrected hit rate in the mnemonic similarity task, source memory accuracy in the acquired equivalence task, corrected hit rate in the face recognition task, and object-color associative memory from the colored objects task (average color error, reverse scored such that higher error yields lower score). The grouping of measures into composite scores was based on prior empirical and theoretical work attributing the performance on those measures to memory specificity or generalization processes respectively (Bowman & Zeithamova, 2025; Taylor et al., 2021; Zeithamova & Bowman, 2020; Zeithamova & Preston, 2017). We reasoned that using multiple measures for each construct—rather than relying on a single task to operationalize each memory process—would better reflect the multiple facets of generalization and specificity. In the results section, we first report basic measures from DRM and all predictor tasks, all pairwise correlations, and finally the main analysis predicting DRM scores from individual differences in memory specificity and generalization abilities using multiple regression.

## Results

### Overview of individual measures

Descriptive statistics (mean, standard deviation) and reliability of each measure is summarized in Table 1. Theoretical chance performance is 0.5 in acquired equivalence and cartoon animal categorization, 0.33 in face categorization and associative inference, 0.25 in acquired equivalence source memory test, and 90 degrees in colored objects. For all other measures, chance is zero. Participants performed above chance on all measures (all t > 39, p < 0.001). Please note that the chance in source memory test is affected by response bias and/or awareness that “study only” response is never correct and is provided for orientation only.

### True and false recall in the Deese, Roediger, and McDermott Task

**Figure 6:**
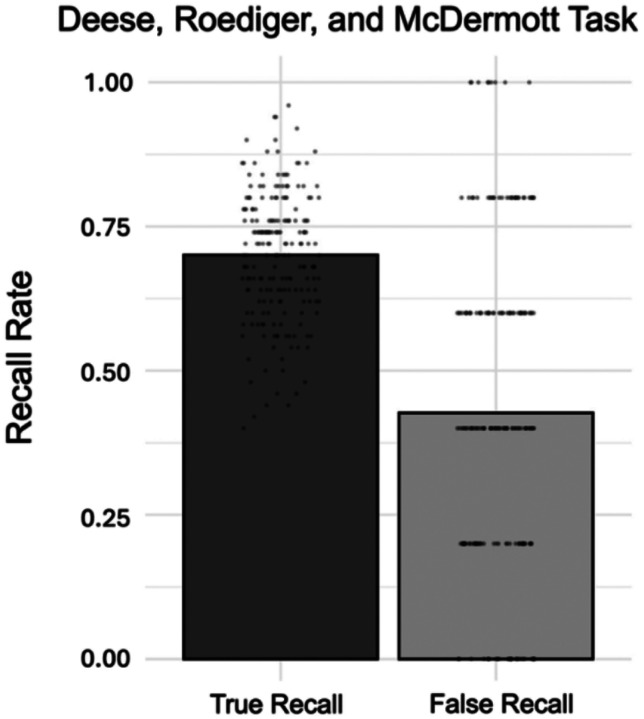
DRM paradigm performance overlaid with individual scores

Mean true recall and false recall rates are presented in Fig. 6. Consistent with prior work, participants frequently recalled not only the words presented, but also the critical lures. Specifically, participants on average recalled 38.7 out of the 50 presented words and falsely recalled 2.13 out of 5 critical lures. Importantly, there was a great degree of variability in both true recall and false recall rates, making it possible to investigate how memory abilities predict individual differences in susceptibility to false memory. True and false recall were negatively correlated with each other (r = − 0.30, p < 0.001) indicating that lower or higher false recall is unlikely driven by lower or higher total verbal output.

#### Performance on generalization measures

**Figure 7:**
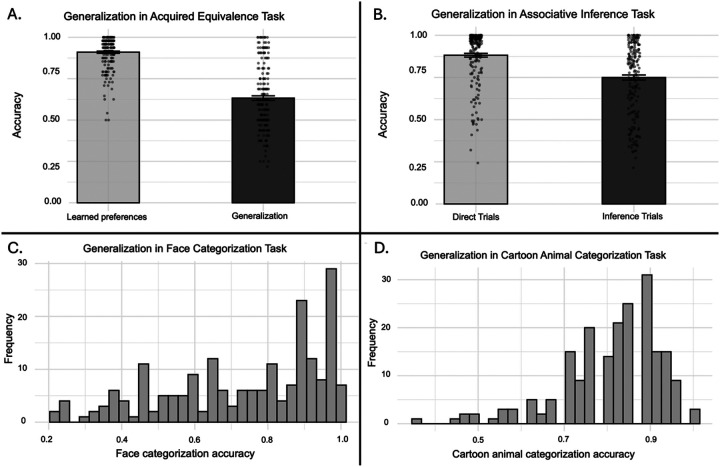
Results from Generalization Measures Note. Measures of generalization. A. Accuracy on learned trials and generalization tendency on generalization test trials in the acquire equivalence task. B. Accuracy on direct associations and inference trials in the associative inference task. C. Distribution of proportion correct (accuracy) in the face categorization task. D. Distribution of proportion correct (accuracy) in the cartoon animal categorization task.

Accuracy for trained associations and the tendency to generalize across related faces (acquired equivalence) from the acquired equivalence task are presented in Fig. 7a. The acquired equivalence score for individual differences analysis was calculated from trials with remembered premise pairs (de Araujo Sanchez & Zeithamova, 2023; Zeithamova, Schlichting, et al., 2012). The accuracy on directly learned and inference trials in the associative inference task is presented in Fig. 7b. The main measure of interest was accuracy on inference associations, limited to trials where participants correctly remembered the corresponding direct pairs. Histograms of categorization scores from the test phases of the face categorization task and cartoon animal categorization are presented in Fig. 7c,d. Accuracy on all measures of generalization was significantly above chance (Table 2, Fig. 7; all t > 60.46, p < 0.001, one-tailed one-sample t-tests comparing to chance). Reliability (Table 2) was good to excellent for all measures except cartoon animal generalization task, which was somewhat lower.

#### Performance on memory specificity measures

**Figure 8:**
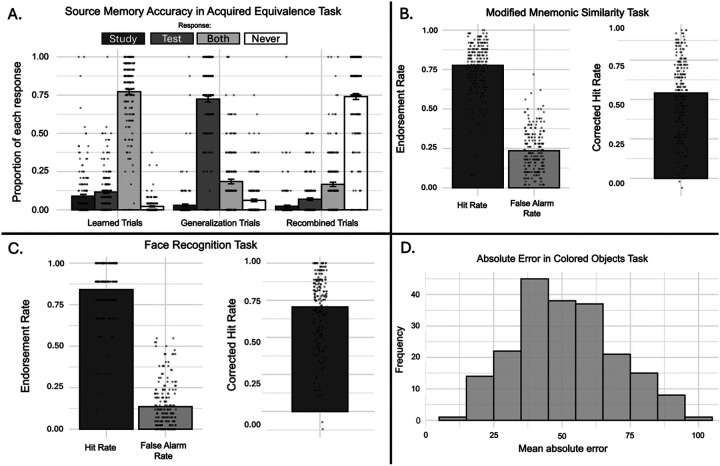
Results from Specificity Measures Note. Measures of memory specificity. A. Proportion of each response in the acquired equivalence task, grouped by trial type. B. Hit rate, false alarm rate, and corrected hit rate in the animal and tool recognition task. C. Hit rate, false alarm rate, and corrected hit rate in the face categorization task D. Absolute error in colored objects task.

Results from the measures of memory specificity are presented in Fig. 8. The proportion of each response to each source memory test trial type for the acquired equivalence task is presented in Fig. 8a. Participants most commonly selected the correct option for each trial type: for trained trials, “both study and test” was the most frequent answer, for untrained trials, “test” was the most frequent answer, and for recombined trials, “never” was the most frequent answer. Nevertheless, large individual differences emerged as well. For the purpose of relating individual differences in source memory to DRM, we calculated for each participant the overall source memory accuracy (M = .75, SD = .18). The overall hit rate, false alarm rate, and corrected hit rate in the mnemonic similarity task and face recognition task are presented in Fig. 8b and 8c, respectively. Individual scores are overlaid to exemplify individual differences in performance in these tasks. Finally, the histogram of mean absolute errors in the colored objects task is presented in Fig. 8d. We also calculated item recognition score for each participant (M = 0.83, SD = 0.14), which was highly correlated with color memory precision (r = 0.76, p < 0.001). We did not include this second memory measure in the composite score as to not give extra weight to this task. Reliability of all specificity measures was moderate to good (Table 2).

### Predicting false recall from memory specificity and generalization abilities

Zero-order correlations from all pairs of task measures and between each measure and specificity and generalization composite are presented in Table 3. We first examined the extent to which individual differences in the false recall on the DRM task were related to individual differences in memory specificity and generalization, as measured by their respective composite scores. Using Pearson’s correlation between DRM false alarm rate and specificity composite, we found that false recall was negatively correlated with individual differences in memory specificity (r= −0.22, p = 0.002), consistent with prior findings. Notably, we found that false recall was also negatively correlated with generalization (r = −0.15, p = 0.03). This finding is in the opposite direction than hypothesized, indicating that participants more successful on generalization tasks were *less* prone to false recall than participants less successful on generalization tasks. As can be seen in Table 3, the correlation between false recall and generalization was also numerically negative for each of the 4 individual generalization measures.

**Figure 9:**
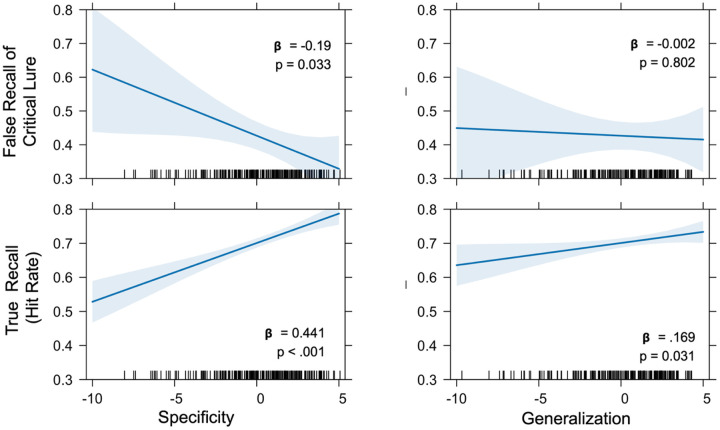
The relationship between generalization and specificity composite scores, and true and false memory measures from DRM Note. Two multiple regressions are visualized, with memory specificity and generalization as predictors and DRM false recall or true recall as an outcome. Top row: false recall of critical lures was negatively related to specificity, and unrelated to generalization. Bottom row: both specificity and generalization predict unique variance in DRM true recall. Tick lines on the bottom of each plot represent individual scores.

Since generalization decisions can be, to some degree, supported by memory for specific events (Zeithamova & Bowman, 2020), we may not be observing a trade-of between generalization ability and false memory because some variability in generalization scores is driven by variability in memory specificity. The composite scores of individual differences in memory specificity and generalization were indeed correlated (r= .66, p < .001). Results of a multiple regression analysis testing the unique contributions of each memory ability while controlling for each other is presented in Fig. 9. We found that memory specificity was a significant predictor of false memory (**β** = −0.19, p = 0.03), with greater specificity score being associated with lower false recall in DRM. Generalization scores did not account for any unique variance in DRM false recall (**β** = −0.002, p = 0.80). Thus, our results align with the idea that DRM false alarms are driven by a lack of specificity in the memory trace, but provide no evidence that individuals who generalize well across experiences would be more prone to false recall in the DRM task.

### Predicting true recall from memory specificity and generalization abilities

Next, we examined how memory for specific details and generalization related to true recall in DRM (Fig. 9, bottom row). We expected that better performance on memory specificity tasks would be associated with better DRM true recall but were interested in whether the ability to generalize and extract commonalities across experience provides any additional benefit, given the common theme within each DRM word list. Simple correlations indicated that true recall was positively correlated with composite scores of both memory specificity (r = 0.55, p < 0.001) and memory generalization (r = 0.46, p < 0.001). A multiple regression with memory specificity and generalization as predictors confirmed that specificity ability was a significant predictor of DRM true recall (**β** = 0.44, p < 0.001). Notably, generalization ability remained a significant predictor of true recall even after taking memory specificity into account (**β** = 0.169, p = 0.031). Thus, greater generalization ability was associated with better true recall of list items, without increasing false memory.

## Discussion

The present study sought to understand how individual differences in false recall rates in the DRM paradigm relate to individual differences in memory specificity and generalization, derived from multi-measure assessments of these abilities. Consistent with our hypothesis and prior work, we found that individuals with high memory specificity score showed lower tendency to false recall. In contrast, we found no evidence for the prediction that stronger generalization would lead to increased false memory; the relationship between false recall and generalization was negative and was reduced near zero once memory specificity was taken into account. These finding inform current theories of false memory and nuance the view of false memory as a flip side of an adaptive generalization process.

The current finding showing that greater memory specificity is associated with lower false recall align with theories and previous work emphasizing the lack of memory specificity in the formation of false memory, including fuzzy trace theory (Brainerd & Kingma, 1984; Brainerd & Poole, 1997; Brainerd & Reyna, 2002; Reyna, 2012) and spreading activation and source monitoring errors (Johnson, 1997; Johnson et al., 1993; Lindsay & Johnson, 1989; Mitchell & Johnson, 2000; Roediger et al., 2001). Other previous work has also linked limited memory specificity with greater instances of false memory on the group and individual differences levels (Ball, Robison, et al., 2022; Unsworth & Brewer, 2010). False recognition is shown to be reduced when participants are given item-specific encoding instructions (Mccabe et al., 2004). On the individual differences level, variability in source monitoring ability is linked with heightened rates of false recognition and recall (Ball, Robison, et al., 2022; Unsworth & Brewer, 2010). Thus, false memory appears reliably linked to a lack of specificity in memory representations.

Interestingly, among the memory specificity measures that contributed to the composite score, the two tasks that correlated the highest with DRM false recall were both measuring perceptual memory specificity, such as precise color-object association or discriminating two visually similar images from the same semantic category (e.g., two images of cats). These findings replicate prior findings connecting perceptual and semantic false recognition (Ball, Robison, et al., 2022; Ball, Wiemers, et al., 2022; West et al., 2025, 2026). Moreover, while prior work linking conceptual and perceptual false memories typically used DRM false *recognition* measures, here we provide a direct link between the precision of perceptual memory and semantic false *recall*. It is rather intriguing that participants who tended to confuse one cat with a similar looking cat in memory were also those who tended to falsely recall critical lures, such as spontaneously coming up with the word “sweet” after being exposed to conceptually related words. This finding is notable because it expands the previous link between semantic and perceptual false recognition to recall, a generative process minimally affected by a simple response bias.

The finding that false memory relates to a lack of specificity in memory trace aligns with a view of false memory as a type of memory failure. Others view false memory as a necessary feature of a system optimized for prediction rather than verbatim recall, where false memory may reflect an adaptive process of abstraction of a common theme across experiences (Bowman et al., 2021; Pardilla-Delgado & Payne, 2017; Schlichting & Preston, 2015; Shohamy & Wagner, 2008). Based on the idea that false memory and generalization are two sides of the same coin, both driven by the same memory integration process, we hypothesized their positive correlation at the individual differences levels such that those most successful in integrating across experiences to derive new knowledge being also most likely to confuse derived knowledge with actual experience. Contrary to this view, we found no evidence that false memories would track generalization success across participants. Simple correlations indicated a *negative* correlation between generalization and false recall, with better generalization associated with *lower* tendency towards false recall. In a multiple regression analysis controlling for memory specificity, generalization performance did not account for any unique variance in false recall rates. As most tasks targeted at assessing generalization also inherently involve the memory for specific learning instances (Zeithamova & Bowman, 2020), these results suggest that the negative relationship between generalization and false recall observed in a simple correlation analysis was likely driven by their shared relationship with memory specificity. The lack of relationship between generalization and false recall in DRM suggests that the presumed extraction of a ‘gist’ driving false recall in DRM may not reflect the same processes as the constructive use of prior knowledge, as measured by generalization tasks.

While prior work has consistently linked false memory to a lack of memory specificity, evidence for the hypothesized link to generalization has been mixed. Aligned with current results, several prior studies showed either negative or no relationship between generalization and false memory (Banino et al., 2016; Bowman et al., 2021; de Araujo Sanchez & Zeithamova, 2023; Varga et al., 2019 Experiment 1). Here, we extend this work by including a measure of memory specificity, which is known to contribute to both and might have contributed to prior findings of a negative relationship. The lack of a trade-off further aligns with the proposals that information may be encoded at multiple levels of specificity in parallel, with integration of information across experience in support of generalization being possible without a cost to memory for individual experiences (Banino et al., 2016; Bowman & Zeithamova, 2025; de Araujo Sanchez & Zeithamova, 2023; Zeithamova & Bowman, 2020).

Nevertheless, other studies suggested that generalization may contribute to false memory under some circumstances. One set of studies observed a relationship between generalization (measured via associative inference) and false memory for contextual information on a trial by trial level (Carpenter & Schacter, 2017, 2018). Interestingly, false memory did not arise by mere exposure to materials that could be generalized nor did it predict subsequent generalization success. Instead, participants showed false memory selectively *after they were tested* on generalization, and only emerged for successfully generalized trials. Thus, it is possible that a relationship between generalization and false memory may emerge when initially separate experiences are connected in memory in response to task demand, and that this false memory creation process may be distinct from the type of stable individual differences in generalization and false recall measured here.

Furthermore, others found in some paradigms that individuals with higher inference abilities (Varga et al., 2019; Warren et al., 2014) and greater creative use of prior knowledge (Thakral et al., 2021, 2023) may be more likely to experience instances of false memory. For example, a study by Warren and colleagues showed that false recall is reduced in prefrontal patients (Warren et al., 2014) who also exhibit generalization impairments (Spalding et al., 2015). Varga and colleagues found some evidence of a relationship between false recognition and generalization in an experiment which emphasized novel inferences as positive outcomes rather than false memories (Varga et al., 2019). However, when false memory was tested traditionally, no relationship was found between DRM false recognition and generalization, consistent with current results (Varga et al., 2019: Experiment 1). Thus, even if a link between individual differences in generalization and false memory exists, this relationship appears conditional rather than robust.

The present study highlights a positive relationship between generalization and *true* recall. This relationship held even after controlling for memory specificity, indicating that individual differences in generalization account for unique variance in true recall scores. As DRM lists share a common theme rather than being arbitrary list of words, the link between generalization and true recall aligns well with prior work on factors affecting memory retrieval. For example, lists organized by categories are remembered better than disorganized lists (Tulving, 1962) and encoding meaning promotes memory (Begg & Wicklegren, 1974; Bransford & Johnson, 1972; Craik & Lockhart, 1972; Craik & Tulving, 1975). Participants who are better in extracting regularities among experiences may be more likely to notice the DRM list organization and meaning, which may in turn facilitate their recall. Encoding the individual words in the context of extracted schema of the list may also facilitate encoding of individual information (Bransford & Johnson, 1972; Craik & Tulving, 1975). Here, we demonstrate novel evidence of these classic encoding principles at the level of individual differences. Participants with greater tendency to extract commonalities across experiences, as measured by greater generalization success across a range of tasks, are also more likely to extract a common these across DRM list members, which in turn facilitates encoding and guides retrieval. Taken together, these findings suggest that there is a critical component of generalization that facilitates encoding and recall of information with internal structure, without a cost to memory fidelity.

In conclusion, the current study assessed how individual differences in false recall relate to the ability to extrapolate across events versus the ability to remember differentiating details. The results pointed to a lack of detail in memory trace is a main contributor to DRM false recall, an idea aligning with previous findings and multiple established theories of false memory. Contrary to our prediction, we found no evidence that increased generalization ability would go hand in hand with increased false memory. These findings inform theories of the constructive nature of memory and reinforce the need for a nuanced approach to the adaptive view of false memory.

## Figures and Tables

**Table 1:** Battery of memory specificity and generalization tasks administered, and the measures of interest extracted from each task

Task	Specificity measure	Generalization measure
Acquired equivalence	Source memory	Acquired equivalence
Associative inference	-----	Associative inference
Categorization (faces)	Old/new recognition	Categorization accuracy
Categorization (cartoon animals)	-----	Categorization accuracy
Colored objects	Precision of object-color association	-----
Mnemonic similarity task	Old/new recognition	-----

**Table 2:** Descriptives and reliability for all measures of interest.

Task Name	Mean	SD	Reliability
True recall in the DRM task	0.70	0.10	0.61
False recall in the DRM task	0.43	0.28	0.47
Acquired equivalence accuracy	0.72	0.27	0.75
Associative inference accuracy	0.75	0.22	0.92
Face categorization accuracy	0.74	0.22	0.86
Cartoon animal categorization accuracy	0.81	0.11	0.54
Source memory accuracy in acquired equivalence	0.75	0.18	0.77
Corrected hit rate in mnemonic similarity	0.54	0.24	0.81
Absolute error in colored objects	51.4	18.3	0.72
Corrected hit rate in face recognition	0.71	0.24	0.75

*Note*. SD = standard deviation. Reliability: Split-half reliability calculated with Spearman-Brown correction.

**Table 3:** Zero-order correlations for all pairs of measures

Tasks	DRM FA	DRM HR	AE	AI	FCAT	ACAT	AESM	MST	FREC	CO
DRM false recall	---									
DRM true recall	−0.30***	---								
Acquired Equivalence	−0.09	0.28***	---							
Associative Inference	−0.16*	0.43***	0.37***	---						
Face Categorization	−0.11	0.30***	0.36***	0.31***	---					
Animal Categorization	−0.07	0.29***	0.26***	0.36***	0.30***	---				
AE Source Memory	−0.11	0.28***	0.35***	0.31***	0.23***	0.17*	---			
Mnemonic similarity	−0.20**	0.47***	0.37***	0.57***	0.34***	0.30***	031***	---		
Face Recognition	−0.07	0.27***	0.30***	0.31***	0.28***	0.21**	0.22**	0.28***	---	
Colored Objects Absolute Error	−0.21**	0.51***	0.38***	0.48***	0.32***	0.26***	0.24***	0.53***	0.29***	---
Specificity composite	−0.21**	0.55***	.50***	0.60***	0.42***	0.34***	0.64***	0.76***	0.47***	0.74***
Generalization composite	−0.15*	0.46***	0.71***	0.73***	0.70***	0.68***	0.375***	0.56***	0.22***	−0.51***

Note.

* p<.05,

** p<.01,

*** p<.001.

Full names listed in column one and abbreviated in row one. AE = acquired equivalence, AI = associative inference, FCAT = face categorization, ACAT = animal categorization, AESM = acquired equivalence source memory, MST = mnemonic similarity task, FREC = face recognition, CO = colored objects. Correlation between specificity and generalization composite scores was r = 0.66.

## Data Availability

The data, task codes, and analysis scripts used to support the findings of this study are openly accessible at: https://osf.io/3245n
